# Plasma Biomarkers and Disease Prognosis in Mild Cognitive Impairment with Lewy Bodies

**DOI:** 10.1002/mds.30181

**Published:** 2025-03-29

**Authors:** Paul C. Donaghy, Jahfer Hasoon, Calum A. Hamilton, Joanna Ciafone, Rory Durcan, Nicola Barnett, Kirsty Olsen, Sarah Lawley, Gemma Greenfinch, Michael Firbank, Amanda Heslegrave, Henrik Zetterberg, Louise Allan, John T. O'Brien, John‐Paul Taylor, Alan J. Thomas

**Affiliations:** ^1^ Translational and Clinical Research Institute and NIHR Newcastle Biomedical Research Centre Newcastle University Newcastle upon Tyne UK; ^2^ Department of Psychiatry University of California, San Diego San Diego California USA; ^3^ Institute of Nuclear Medicine University College London Hospitals NHS Foundation Trust London UK; ^4^ UK Dementia Research Institute at UCL London UK; ^5^ Department of Neurodegenerative Disease UCL Institute of Neurology London UK; ^6^ Department of Psychiatry and Neurochemistry, Institute of Neuroscience and Physiology The Sahlgrenska Academy at the University of Gothenburg Mölndal Sweden; ^7^ Clinical Neurochemistry Laboratory Sahlgrenska University Hospital Mölndal Sweden; ^8^ Hong Kong Center for Neurodegenerative Diseases Hong Kong China; ^9^ Wisconsin Alzheimer's Disease Research Center, University of Wisconsin School of Medicine and Public Health University of Wisconsin‐Madison Madison Wisconsin USA; ^10^ Centre for Research in Ageing and Cognitive Health University of Exeter Exeter UK; ^11^ Department of Psychiatry, School of Clinical Medicine University of Cambridge Cambridge UK

**Keywords:** Alzheimer's disease, dementia with Lewy bodies, mild cognitive impairment, plasma biomarkers, prognosis

## Abstract

**Background:**

Little is known about the prognostic value of plasma biomarkers in mild cognitive impairment with Lewy bodies (MCI‐LB).

**Objectives:**

To investigate the association of four plasma biomarkers with disease progression in MCI.

**Methods:**

Plasma amyloid‐beta (Aβ)_42/40_, glial fibrillary acidic protein (GFAP), neurofilament light (NfL), and phosphorylated tau 181 (pTau181) were measured at baseline in a longitudinal MCI cohort (n = 131).

**Results:**

Baseline plasma NfL was associated with increased risk of dementia/death in the entire cohort. In MCI‐LB, baseline plasma NfL, GFAP, and pTau181 were associated with increased risk of dementia/death and increased cognitive decline measured by the Addenbrooke's Cognitive Examination‐Revised.

**Conclusions:**

pTau181, GFAP, and NfL are associated with more rapid disease progression in MCI‐LB and, with further validation, could be useful to support prognosis and stratification for clinical practice and treatment trials. Further work, including clinicopathological studies, is needed to understand the biological correlates of these markers in MCI‐LB. © 2025 The Author(s). *Movement Disorders* published by Wiley Periodicals LLC on behalf of International Parkinson and Movement Disorder Society.

Blood‐based biomarkers are well established in dementia research and are entering clinical use. A blood biomarker that could stratify people with mild cognitive impairment with Lewy bodies (MCI‐LB) based on likely future cognitive decline would be useful in clinic and for the stratification of participants in clinical trials.^1^

The presence of Alzheimer's disease (AD) pathology measured by cerebrospinal fluid (CSF) or positron emission tomography (PET) has been associated with more rapid cognitive decline in dementia with Lewy bodies (DLB).^2^, ^3^ The prognostic value of plasma biomarkers has been investigated in DLB,^4^, ^5^ but little is known about the predictive value of these biomarkers in MCI‐LB.

The objectives of this study were to investigate the association of four plasma biomarkers – amyloid‐beta (Aβ)_42/40_, glial fibrillary acidic protein (GFAP), neurofilament light (NfL), and phosphorylated tau 181 (pTau181) – and disease progression in MCL‐LB measured by: (1) conversion to dementia/death and (2) cognitive performance. We have previously reported that pTau181, GFAP, and NfL are raised in MCI‐LB and MCI‐AD compared with controls and that pTau181 and GFAP are lower in MCI‐LB than MCI‐AD.^6^ In a previous study from this cohort, with shorter follow‐up, cases were binarized as low or high pTau181 and rates of decline in cognitive test scores did not differ between the groups.^7^ Here we report plasma GFAP, NfL, and Aβ_42/40_ in addition to pTau181 over a longer period of follow‐up. The addition of longer follow‐up over a mean of 3 years allows us to report the association between plasma biomarker levels and conversion to dementia or death. We hypothesised that higher baseline plasma pTau181, GFAP, and NfL would be associated with a greater hazard of dementia/death and more rapid cognitive decline.

## Methods

### Participants

Participants aged ≥60 years with MCI were recruited from February 2013 to September 2019 as outlined previously.^8^, ^9^ All participants gave their written informed consent to take part in the study. The study received ethical approval from the National Research Ethics Service Committee North East–Newcastle & North Tyneside 2 (15/NE/0420, 12/NE/0290).

### Clinical Assessment and Diagnosis

Participants had a detailed clinical assessment at baseline and annually where possible including the Addenbrooke's Cognitive Examination‐Revised (ACE‐R) and Mini‐Mental State Examination (MMSE). All participants were included in the longitudinal analysis, even if clinical follow‐up had not been completed, as participants had consented to electronic health record review and, therefore, time to death could still be ascertained.

Details of the diagnostic classification of participants have been included in previous publications.^8^, ^9^ At each visit, a three‐person diagnostic panel (P.C.D., A.J.T., J.‐P.T.) determined whether the participant had MCI or dementia, and the presence of core clinical features of MCI‐LB and DLB. Participants were classified as probable/possible MCI‐LB at baseline if their final diagnosis after longitudinal follow‐up was either probable/possible MCI‐LB^10^ or they had progressed to probable/possible DLB.^11^ Participants were classified as MCI‐AD at baseline if their final diagnosis was either MCI‐AD^12^ or AD dementia.^13^, ^14^

### Blood Sampling and Analysis

Venous blood was collected by venepuncture in EDTA tubes at baseline assessment. Plasma was isolated by centrifugation, aliquoted, and stored at −80°C. Plasma samples were analyzed by the UK Dementia Research Institute Biomarker Laboratory using single‐molecule array (Simoa) with the Quanterix Simoa Human Neurology 4‐Plex E and HD1 Analyzer for Aβ_40_, Aβ_42_, GFAP, and NfL and the Quanterix Simoa pTau181 Advantage kit and HD‐X Analyzer (Quanterix, Billerica, MA, USA). For each analyte, all samples were analyzed with the same batch of reagents, at the same time as previously reported.^6^, ^7^ The mean inter‐assay percentage coefficients of variation (CVs) were 3.80% for Aβ_40_, 3.17% for Aβ_42_, 6.13% for GFAP, 4.24% for NfL, and 2.41% for pTau181.

### Statistical Analysis

Statistical analysis was completed using IBM SPSS Version 29.0.1.0 and MATLAB R2022a. pTau181, GFAP, and NfL concentrations were base 10 log transformed.

Cox proportional hazards models were used to calculate hazard ratios for conversion to dementia or death with correction for age, sex, years in education, and MMSE. Age and years in education were normalized to the total mean, and MMSE and blood results were standardized with respect to the control mean. The proportional hazard assumption was assessed using scaled Schoenfeld residuals. The Aβ_42/40_ ratio was inversed and so a greater value represents greater abnormality to allow for direct comparison of hazard ratios.

Linear regression models were undertaken with annualized cognitive score change as the dependent variable and individual plasma biomarker levels alongside age, education, sex, and baseline cognitive score as covariates. We analyzed the combined cohort as well as the MCI‐AD and probable MCI‐LB groups separately. The possible MCI‐LB group was included in the combined cohort only, as the diagnosis of these cases is less certain.

## Results

### Participants and Duration of Follow‐Up

A total of 161 participants completed baseline assessment, of which 149 completed at least one follow‐up assessment (Table S1). The MCI‐AD and probable MCI‐LB groups were well‐balanced in age, years in education, baseline MMSE, and duration of follow‐up. The probable MCI‐LB had a lower proportion of females compared with MCI‐AD (22% vs. 60%). Group comparisons in plasma biomarkers have been reported previously.^6^

### Prediction of Conversion to Dementia or Death

In the entire MCI cohort, 72/131 participants (55%) converted to dementia or died. Median (interquartile [IQR]) time to dementia or death was 2.0 (1.0–3.1) years. Baseline NfL was associated with a greater hazard of conversion to dementia or death (Table 1).

**TABLE 1:** Plasma biomarker hazard ratios for dementia or death in mild cognitive impairment.

Plasma biomarker	HR (95% CI)	*P*
All MCI (n = 120)		
Aβ_42/40_ ^a^	1.06 (0.83–1.35)	0.636
NfL	**1.58 (1.17–2.11)**	**0.002**
GFAP	1.27 (0.95–1.70)	0.107
pTau181	1.21 (0.97–1.51)	0.093
Probable MCI‐LB (n = 62)		
Aβ_42/40_ ^a^	1.23 (0.94–1.60)	0.130
NfL	**1.74 (1.22–2.48)**	**0.002**
GFAP	**1.74 (1.21–2.50)**	**0.003**
pTau181	**1.50 (1.11–2.02)**	**0.009**
MCI‐AD (n = 43)		
Aβ_42/40_ ^a^	0.67 (0.37–1.20)	0.180
NfL	1.29 (0.71–2.33)	0.399
GFAP	0.73 (0.41–1.28)	0.267
pTau181	1.05 (0.68–1.64)	0.825

*Note*: Hazard ratio (95% confidence interval) and significance for the coefficient from the Cox proportional hazards model for the entire cohort, probable MCI‐LB, and MCI‐AD. Model includes age, years in education, sex, and baseline Mini‐Mental State Examination (MMSE) as covariates.

Abbreviations: HR, hazard ratio; CI, confidence interval; MCI, mild cognitive impairment; Aβ, amyloid‐beta 42/40 ratio; NfL, neurofilament light; GFAP, glial fibrillary acidic protein; pTau181, phosphorylated tau 181; MCI‐LB, mild cognitive impairment with Lewy bodies; MCI‐AD, mild cognitive impairment due to Alzheimer's disease.

^a^ Results have been reversed to allow for direct comparison of hazard ratios. Significant results are highlighted in bold type (*P* < 0.05).

In probable MCI‐LB, 41/63 participants (65%) converted to dementia or died. Median (IQR) time to dementia or death was 1.4 (1.0–2.5) years. Baseline NfL, GFAP, and pTau181 were all associated with an increased hazard of conversion to dementia or death.

**FIG 1:**
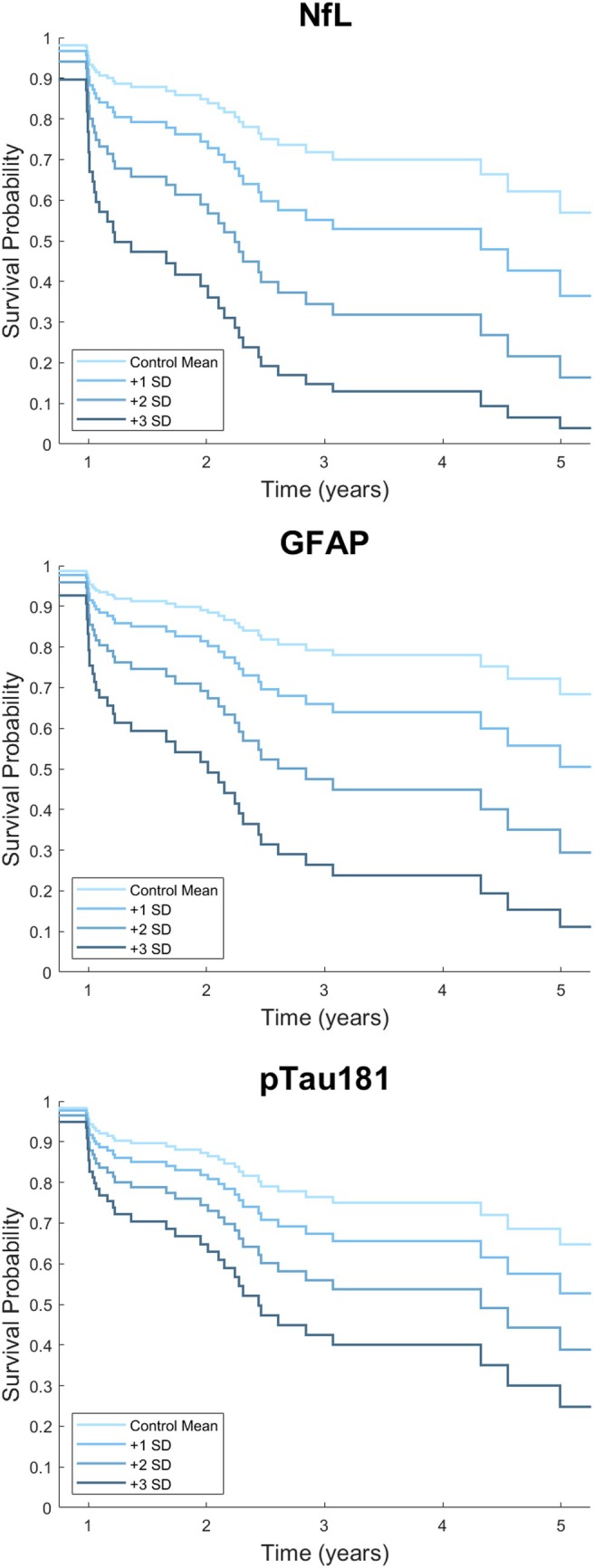
Modelled survival plots for plasma biomarkers for probable mild cognitive impairment with Lewy bodies (MCI‐LB). The lines represent the estimated expected survival for blood results at the control mean (lightest line) and increasing abnormality by 1, 2, and 3 standard deviations from the control mean represented by increasing darkness of the lines. NfL, neurofilament light; SD, standard deviation; GFAP, glial fibrillary acidic protein; pTau181, phosphorylated tau 181. [Color figure can be viewed at wileyonlinelibrary.com]

In MCI‐AD, 21/47 participants (45%) converted to dementia or died. Median (IQR) time to dementia or death was 2.2 (1.1–3.5) years. No marker was significant for these outcomes in MCI‐AD. Figure 1 displays modeled survival curves showing the potential effect of increasingly abnormal plasma biomarker results on expected survival in probable MCI‐LB.

### Prediction of Cognitive Decline Measured by ACE‐R and MMSE

Increased plasma pTau181 was associated with greater decline in ACE‐R and MMSE in the entire MCI cohort. In probable MCI‐LB, increased pTau181, GFAP, and NfL were associated with greater decline in ACE‐R, but not MMSE. In MCI‐AD, no markers were associated with rate of change in MMSE or ACE‐R (Tables S2 and S3).

## Discussion

### Plasma Biomarkers and Disease Progression in DLB and MCI‐LB

This article demonstrates that plasma GFAP, NfL, and pTau181 are associated with the risk of conversion to dementia or death in probable MCI‐LB and are associated with cognitive decline measured using the ACE‐R over a mean of 2.7 years. To our knowledge, the current cohort is the only one to have investigated the link between plasma biomarkers and cognitive decline in MCI‐LB.

In the near future, plasma biomarkers may be used as part of the diagnostic process in dementia and MCI. Our findings suggest that such markers may also help patients with MCI‐LB by allowing clinicians to provide an estimation of the likely rate of decline or risk of dementia in the future. Prior to this, the findings must be replicated in further MCI‐LB cohorts and validated thresholds for abnormal results need to be developed. Plasma biomarkers may also have a role in clinical trials to select or stratify participants based on predicted decline over time and to enrich trial cohorts with participants who are more likely to progress to dementia.

In a previous publication from this cohort, with shorter follow‐up, cases were binarized as low or high pTau181, and rates of decline did not differ between the groups.^7^ The correlation observed between cognitive decline and plasma pTau181, GFAP, and NfL in the present study (with longer follow‐up) is in keeping with findings in DLB that plasma GFAP, NfL, pTau181, and pTau231 are associated with decline in cognition,^4^, ^5^ although a smaller DLB cohort found no association between these markers and cognitive decline.^15^

### Interpretation of Plasma Biomarkers in MCI‐LB

These results may also provide clues regarding the pathophysiology of cognitive decline in MCI‐LB. However, it is important to consider the evidence base when interpreting the meaning of raised plasma biomarkers, recognizing that the primary source of the signal may be different in AD, where high levels of tau pathology are present, and DLB, where relatively low levels of tau pathology are present, particularly in early disease. One study has demonstrated correlation of GFAP, pTau181, and NfL with amyloid PET, but not tau PET, in MCI‐LB.^16^ GFAP displayed the strongest difference in association between the two PET modalities, having the strongest correlation with amyloid PET (Pearson's partial *r* = 0.68, *P* < 0.001) and no evidence of association with tau PET (*r* = 0.05, *P* = 0.81). In contrast, pTau181 and NfL had some evidence of a relationship with tau PET (*r* = 0.24–0.35), though weaker than their relationship to amyloid PET, and not statistically significant. Current evidence therefore suggests that plasma pTau, GFAP, and NfL are associated with brain amyloid deposition and cognitive decline in MCI‐LB. Further work, including clinicopathological studies, is needed to deepen our understanding of the biological correlates of these markers in MCI‐LB.

### Plasma Biomarkers and Disease Progression in AD

We found no relationship between plasma biomarkers and conversion to dementia/death or cognitive decline in MCI‐AD. pTau181 has been shown to be associated with an increased risk of conversion from MCI to dementia and increased rate of cognitive decline in MCI.^17^, ^18^, ^19^ The association between NfL and GFAP with cognitive decline in MCI is less well established.^17^, ^18^ The absence of an association of pTau181 and disease progression in our cohort may be due to a relatively small number of MCI‐AD cases (n = 43) and a lower number of dementia/death events observed (n = 21).

### Study Strengths and Limitations

We used conversion to dementia/death as our primary outcome measure, as this captures people with rapidly progressive disease who are excluded from analyses that require the completion of a cognitive test at follow‐up assessment. In our secondary analyses, we found correlation between plasma biomarkers and cognition measured by ACE‐R, but not the MMSE, in probable MCI‐LB. This may be because the ACE‐R measures aspects of attention, concentration, and visuospatial function and therefore may be more sensitive to change in MCI‐LB than the MMSE. We had multiple cognitive and plasma measures and did not correct for multiple comparisons, therefore these results must be considered exploratory and confirmed in independent cohorts. We used an annualized rate of cognitive decline, which presumes linear decline, though some participants may not have linear progression. At the time of analysis, these biomarkers did not have validated thresholds for normal/abnormal levels, therefore we were unable to classify participants as amyloid positive or negative. There were few transitions from AD to LB disease or vice versa; therefore, we were unable to investigate the association between plasma biomarkers and these transitions. We used pTau181, but there is some evidence that pTau231 may be a better marker of cognitive progression in DLB,^5^ and recent data suggest that pTau217 may be slightly better than the other pTau markers for detecting the neuronal reaction to Aβ pathology.^20^ Finally, diagnosis was determined on clinical grounds. Future studies with autopsy confirmation will be important to validate these biomarkers.

## Conclusions

pTau181, GFAP, and NfL are associated with more rapid disease progression in probable MCI‐LB and, with further validation, could be useful to support prognosis and stratification for clinical practice and treatment trials.

## Author Roles

(1) Research Project: A. Design, B. Organization, C. Execution; (2) Statistical Analysis: A. Design, B. Execution, C. Review and Critique; (3) Manuscript Preparation: A. Writing of the First Draft, B. Review and Critique.

P.C.D.: 1A, 1B, 1C, 2A, 2B, 3A, 3B.

J.H.: 1A, 1C, 2A, 2B, 2C, 3A, 3B.

C.A.H.: 1A, 1B, 1C, 2A, 2B, 2C, 3B.

J.C.: 1C, 3B.

R.D.: 1C, 3B.

N.B.: 1C, 3B.

K.O.: 1C, 3B.

S.L.: 1C, 3B.

G.G.: 1C, 3B.

M.F.: 1A, 1C, 3B.

A.H.: 1A, 1C, 3B.

H.Z.: 1A, 1C, 3B.

L.A.: 1A, 1C, 3B.

J.T.O.: 1A, 1C, 3B.

J.‐P.T.: 1A, 1C, 3B.

A.J.T.: 1A, 1B, 1C, 2A, 2C, 3B.

## Financial Disclosures of All Authors (for the Preceding 12 Months)

P.C.D.: Received grants from the Medical Research Council, National Institute for Health and Care Research (NIHR) Newcastle Biomedical Research Centre, Alzheimer's Society, Alzheimer's Research UK, and The Michael J. Fox Foundation; member of the Lewy Body Society Specialist Advisory Committee (unremunerated); and honoraria from the Lewy Body Academy (paid to institution). J.H., C.A.H., J.C., R.D., N.B., K.O., S.L., G.G., M.F.: None. A.H.: Acted as a consultant for Quanterix and Lilly. H.Z.: Is a Wallenberg Scholar and a Distinguished Professor at the Swedish Research Council supported by grants from the Swedish Research Council (#2023–00356, #2022–01018, and #2019–02397), the European Union's Horizon Europe Research and Innovation Programme under Grant Agreement No. 101053962, Swedish State Support for Clinical Research (#ALFGBG‐71320), the Alzheimer Drug Discovery Foundation (ADDF), USA (#201809–2016862), the AD Strategic Fund and the Alzheimer's Association (#ADSF‐21‐831376‐C, #ADSF‐21‐831381‐C, #ADSF‐21‐831377‐C, and #ADSF‐24‐1284328‐C), the European Partnership on Metrology, co‐financed from the European Union's Horizon Europe Research and Innovation Programme and by the Participating States (NEuroBioStand, #22HLT07), the Bluefield Project, Cure Alzheimer's Fund, the Olav Thon Foundation, the Erling‐Persson Family Foundation, Familjen Rönströms Stiftelse, Stiftelsen för Gamla Tjänarinnor, Hjärnfonden, Sweden (#FO2022‐0270), the European Union's Horizon 2020 Research and Innovation Programme under the Marie Skłodowska‐Curie Grant Agreement No. 860197 (MIRIADE), the European Union Joint Programme–Neurodegenerative Disease Research (JPND2021‐00694), the NIHR University College London Hospitals Biomedical Research Centre, and the UK Dementia Research Institute at University College (UCL) (UKDRI‐1003); and has served on scientific advisory boards and/or as a consultant for AbbVie, Acumen, Alector, Alzinova, ALZpath, Amylyx, Annexon, Apellis, Artery Therapeutics, AZTherapies, Cognito Therapeutics, CogRx, Denali, Eisai, LabCorp, Merry Life, Nervgen, Novo Nordisk, Optoceutics, Passage Bio, Pinteon Therapeutics, Prothena, Quanterix, Red Abbey Labs, reMYND, Roche, Samumed, Siemens Healthineers, Triplet Therapeutics, and Wave; has given lectures sponsored by Alzecure, BioArctic, Biogen, Cellectricon, Fujirebio, Lilly, Novo Nordisk, Roche, and WebMD; and is a co‐founder of Brain Biomarker Solutions in Gothenburg AB (BBS), which is a part of the GU Ventures Incubator Program (outside the submitted work). L.A.: Received research grants from NIHR, Medical Research Council (MRC), Economic and Social Research Council (ESRC), Alzheimer's Society, and Parkinson's UK. J.T.O.: Acted as a consultant for TauRx, Novo Nordisk, Biogen, Roche, Lilly, GE Healthcare, and Okwin; and has received grant or academic funding in kind support from Avid/Lilly, Merck, UCB, and Alliance Medical. J.‐P.T.: Received grants from the NIHR Newcastle Biomedical Research Centre, Alzheimer's Society, Alzheimer's Research UK, The Michael J. Fox Foundation, and NIHR; is a member of the Lewy Body Society Specialist Advisory Committee (unremunerated); has received speaker fees from the Lewy Body Academy, Bial Pharmaceutical, and GE Healthcare; and has undertaken consultancy for EIP Pharma. A.J.T.: None.

## Supporting information


**Table S1.** Group demographics and plasma biomarker results.
**Table S2.** Association between plasma biomarkers and annualized cognitive score change in Addenbrooke's Cognitive Examination‐Revised (ACE‐R) based on first and last measures recorded.
**Table S3.** Association between plasma biomarkers and annualised cognitive score change in Mini‐Mental State Examination (MMSE) based on first and last measures recorded.

## Data Availability

Data and blood samples are available through Dementias Platform UK (www.dementiasplatform.uk).
