# Supplementary material for: Plasma Biomarkers and Disease Prognosis in Mild Cognitive Impairment with Lewy Bodies
Source: Mov Disord. 2025 Mar 29;40(6):1200–5. doi: 10.1002/mds.30181 (PMC12160992; doi:10.1002/mds.30181)
Supplement: Supplementary file 1 — Table S1. Group demographics and plasma biomarker results. Table S2. Association between plasma biomarkers and annualized cognitive score change in Addenbrooke's Cognitive Examination‐Revised (ACE‐R) based on first and last measures recorded. Table S3. Association between plasma biomarkers and annualised cognitive score change in Mini‐Mental State Examination (MMSE) based on first and last measures recorded. [file MDS-40-1200-s001.docx]

**Supplementary Tables**

| **Supplementary Table 1. Group Demographics and plasma biomarker results** | | | | |
| --- | --- | --- | --- | --- |
|  | **Control** | **MCI-AD** | **Poss MCI-LB** | **Prob MCI-LB** |
| **N** | 30 | 47 | 21 | 63 |
| **N with follow-up** | 29 | 43 | 15 | 62 |
| **Age (years)** | 73.8 (7.6) | 76.3 (7.4) | 75.5 (7.3) | 75.5 (7.3) |
| **Sex (Female)** | 8 (26.7%) | 28 (59.6%) | 9 (42.9%) | 14 (22.2%) |
| **Education (years)** | 14.7 (4.0) | 12.4 (3.3) | 12.1 (4.3) | 11.6 (2.7) |
| **Dementia or Death** | 2 (6.7%) | 21 (44.7%) | 10 (47.6%) | 41 (65.1%) |
| **Duration of follow up in years** | 2.4 (1.7) | 2.9 (1.7) | 3.7 (2.7) | 2.7 (1.9) |
| **MMSE** | 28.6 (1.1) | 26.9 (2.1) | 25.6 (2.5) | 26.1 (2.4) |
| **ACE-R** | 93.1 (4.2) | 81.6 (9.6) | 76.1 (10.4) | 79.4 (10.1) |
| **Aβ_42/40_** | .068 (.013) | .063 (.011) | .062 (.007) | .063 (.015) |
| **Log GFAP** | 1.858 (.255) | 2.160 (.235) | 2.058 (.213) | 2.068 (.254) |
| **Log NfL** | 1.241 (.183) | 1.405 (.190) | 1.387 (.162) | 1.353 (.207) |
| **Log pTau181** | .172 (.169) | .408 (.202) | .395 (.181) | .315 (.204) |
| Mean (standard deviation) for continuous variables and count (percent) for categorical variables.  ACE-R, Addenbrooke’s cognitive examination revised version; MMSE, Mini-Mental State Examination; MCI-LB, mild cognitive impairment with Lewy bodies; MCI-AD, mild cognitive impairment due to Alzheimer’s disease; Aβ, amyloid-beta 42/40 ratio ; GFAP, glial fibrillary acidic protein; NfL, neurofilament light; pTau, phosphorylated tau. | | | | |

| **Supplementary Table 2. Association between plasma biomarkers and annualised cognitive score change in ACE-R based on first and last measures recorded.** | | | | |
| --- | --- | --- | --- | --- |
| **All MCI (n=108)** | | | | |
|  | **r** | **p** | **β** | **p** |
| **Aβ_42/40_** | .15 | .131 | .15 (-.04, .34) | .116 |
| **Log GFAP** | -.21 | .033 | -.20 (-.41, .00) | .053 |
| **Log NfL** | -.16 | .110 | -.20 (-.43, .03) | .081 |
| **Log pTau181** | -.24 | .012 | **-.25 (-.45, -.05)** | **.014** |
| **Prob MCI-LB (n=54)** | | | | |
|  | **r** | **p** | **β** | **p** |
| **Aβ_42/40_** | .22 | .113 | .21 (-.07, .49) | .133 |
| **Log GFAP** | -.29 | .035 | **-.34 (-.62, -.05)** | **.023** |
| **Log NfL** | -.25 | .064 | **-.41 (-.72, -.09)** | **.012** |
| **Log pTau181** | -.28 | .041 | **-.30 (-.59, -.01)** | **.045** |
| **MCI-AD (n=40)** | | | | |
|  | **r** | **p** | **β** | **p** |
| **Aβ_42/40_** | .06 | .710 | .09 (-.25, .42) | .606 |
| **Log GFAP** | -.24 | .136 | -.18 (-.57, .22) | .370 |
| **Log NfL** | -.23 | .157 | -.20 (-.65, .24) | .361 |
| **Log pTau181** | -.32 | .044 | -.23 (-.59, .13) | .209 |
| Pearson correlation coefficient and p value are shown for illustration. β coefficient and p value from linear regression with age, education, sex, and baseline cognitive score as covariates. Significant results in linear regression highlighted in bold (p<.05). MCI-LB, mild cognitive impairment with Lewy bodies; MCI-AD, mild cognitive impairment due to Alzheimer’s disease; Aβ, amyloid-beta 42/40 ratio; GFAP, glial fibrillary acidic protein; NfL, neurofilament light; pTau, phosphorylated tau. | | | | |

| **Supplementary Table 3. Association between plasma biomarkers and annualised cognitive score change in MMSE based on first and last measures recorded.** | | | | |
| --- | --- | --- | --- | --- |
| **All MCI (n=109)** | | | | |
|  | **r** | **p** | **β** | **p** |
| **Aβ_42/40_** | .11 | .254 | .11 (-.08, .30) | .252 |
| **Log GFAP** | -.15 | .130 | -.12 (-.33, .09) | .255 |
| **Log NfL** | -.10 | .301 | -.10 (-.33, .13) | .372 |
| **Log pTau181** | -.23 | .017 | **-.22 (-.42, -.01)** | **.037** |
| **Prob MCI-LB (n=55)** | | | | |
|  | **r** | **p** | **β** | **p** |
| **Aβ_42/40_** | .20 | .151 | .19 (-.09, .47) | .171 |
| **Log GFAP** | -.19 | .166 | -.23 (-.53, .07) | .125 |
| **Log NfL** | -.10 | .449 | -.21 (-.54, .12) | .197 |
| **Log pTau181** | -.23 | .088 | -.25 (-.55, .04) | .086 |
| **MCI-AD (n=40)** | | | | |
|  | **r** | **p** | **β** | **p** |
| **Aβ_42/40_** | -.03 | .855 | -.02 (-.36, .33) | .929 |
| **Log GFAP** | -.12 | .475 | .00 (-.43, .44) | .989 |
| **Log NfL** | -.23 | .163 | -.18 (-.62, .26) | .418 |
| **Log pTau181** | -.26 | .105 | -.18 (-.55, .18) | .319 |
| Pearson correlation coefficient and p value are shown for illustration. β coefficient and p value from linear regression with age, education, sex, and baseline cognitive score as covariates. Significant results in linear regression highlighted in bold (p<.05). MCI-LB, mild cognitive impairment with Lewy bodies; MCI-AD, mild cognitive impairment due to Alzheimer’s disease; Aβ_42/40_, amyloid-beta 42/40 ratio; GFAP, glial fibrillary acidic protein; NfL, neurofilament light; pTau, phosphorylated tau. | | | | |
